# The Role of Prebiotics in Modulating Gut Microbiota: Implications for Human Health

**DOI:** 10.3390/ijms25094834

**Published:** 2024-04-29

**Authors:** Suyeon Yoo, Suk-Chae Jung, Kihyuck Kwak, Jun-Seob Kim

**Affiliations:** 1 Department of Nano-Bioengineering, Incheon National University, Incheon 22012, Republic of Korea; 2 Department of Bioengineering, University of Illinois Urbana-Champaign, Urbana, IL 61801, USA; 3 Department of Microbiology and Immunology, Yonsei University College of Medicine, Seoul 03722, Republic of Korea; 4 Institute for Immunology and Immunological Diseases, Yonsei University College of Medicine, Seoul 03722, Republic of Korea; 5 Brain Korea 21 PLUS Project for Medical Sciences, Yonsei University College of Medicine, Seoul 03722, Republic of Korea; 6 Institute for New Drug Development, College of Life Science and Bioengineering, Incheon National University, Incheon 22012, Republic of Korea

**Keywords:** prebiotics, microbiota, intestinal microbiota, human health

## Abstract

The human gut microbiota, an intricate ecosystem within the gastrointestinal tract, plays a pivotal role in health and disease. Prebiotics, non-digestible food ingredients that beneficially affect the host by selectively stimulating the growth and/or activity of beneficial microorganisms, have emerged as a key modulator of this complex microbial community. This review article explores the evolution of the prebiotic concept, delineates various types of prebiotics, including fructans, galactooligosaccharides, xylooligosaccharides, chitooligosaccharides, lactulose, resistant starch, and polyphenols, and elucidates their impact on the gut microbiota composition. We delve into the mechanisms through which prebiotics exert their effects, particularly focusing on producing short-chain fatty acids and modulating the gut microbiota towards a health-promoting composition. The implications of prebiotics on human health are extensively reviewed, focusing on conditions such as obesity, inflammatory bowel disease, immune function, and mental health. The review further discusses the emerging concept of synbiotics—combinations of prebiotics and probiotics that synergistically enhance gut health—and highlights the market potential of prebiotics in response to a growing demand for functional foods. By consolidating current knowledge and identifying areas for future research, this review aims to enhance understanding of prebiotics’ role in health and disease, underscoring their importance in maintaining a healthy gut microbiome and overall well-being.

## 1. Introduction

The human gut is home to more than 1000 species of microorganisms, forming a complex ecological community called the gut microbiota [1]. The human gut microbiota is primarily composed of four phyla: *Firmicutes*, *Bacteroidetes*, *Actinobacteria*, and *Proteobacteria* [2]. A healthy microbiota is high in taxonomic diversity, rich in microbial genes, and often has a stable core of microbes [3]. Next-generation sequencing technologies such as targeted amplicon (e.g., 16S- or 18S rRNA, internal transcribed spacer), shotgun metagenomic, metatranscriptomic, and genomic sequencing are widely applied in gut microbiome research [4]. The number of microorganisms in the gut microbiome is roughly equal to the number of somatic cells in our body, and these microorganisms participate in most metabolic activities in vivo with noticeable effects. Moreover, the gut microbiome contains 600,000 genes [5,6]. This is about 25 times more than the number of genes in the human genome, emphasizing the importance of a highly complex microbiome ecosystem with the potential to impact metabolism and immune function significantly. Gut immune, neural, and endocrine cells are intimately connected and, through host-microbe crossover, together with the gut microbiota, form a highly complex gut ecosystem that contributes to the host’s homeostatic balance [7]. The gut microbiota also provides an essential ability to ferment non-digestible substrates such as dietary fiber. This fermentation promotes the growth of specialized microorganisms capable of producing short-chain fatty acids (SCFAs). The most significant SCFAs produced for human health are acetate, propionate, and butyrate [8]. Prebiotics are substrates that are selectively utilized by host microorganisms to provide health benefits [9]. Prebiotics produce SCFAs through fermentation by gut microorganisms [10]. It has already been known that prebiotics affect human health by modulating the human gut microbiota [11]. This review will discuss prebiotics.

## 2. Prebiotics and Types of Prebiotics

The concept of prebiotics was first introduced in 1995 by Glenn R. Gibson and Marcel Roberfroid, who explained that prebiotics are non-digestible food ingredients that selectively stimulate the growth or activity of one or a limited number of bacteria in the colon, with beneficial effects on the host, thereby improving host health [12]. Later, in 2004, Glenn R. Gibson and Hollie M. Probert proposed three criteria: (i) resisting hydrolysis by mammalian enzymes and gastrointestinal absorption and gastric acidity; (ii) fermented by gut microorganisms; (iii) selectively stimulating the activity and growth of gut bacteria involved in health and well-being [13]. In 2016, the International Scientific Association for Probiotics and Prebiotics (ISAPP) redefined prebiotics as a selectively utilized substrate by host microorganisms conferring a health benefit [9]. The criteria for defining prebiotics are evolving gradually. This review will focus on prebiotics in a broader sense.

### 2.1. Fructans

**Figure 1:**
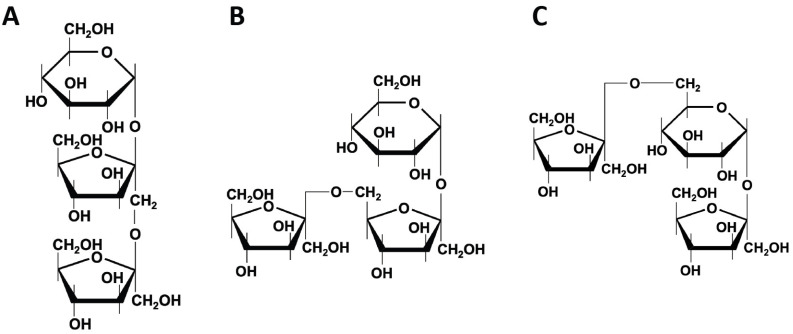
**Chemical structures of fructans.** Differences in fructans structures arise based on the position of fructose addition, leading to the creation of (**A**) 1-kestose (**B**) 6-kestose (**C**) 6G-kestose.

Fructans are natural fructose polymers used for their prebiotic and health-enhancing properties in functional foods. Unlike starch, plant-derived fructans are water-soluble compounds derived directly from sucrose. Different types can be distinguished based on their structure.: inulin (β2→1 linkage), levan (β2→6 linkage), and graminan (β2→1 linkage and β2→6 linkages) [14]. Differences in structure arise based on the position of fructose addition, leading to the creation of 1-kestose, 6-kestose, or 6G-kestose (Figure 1) (Table 1) [15]. Inulin-type fructans, which are polymers of α-linked glucose and β-2,1 linked fructose, are inulin with longer chains (degree of polymerization (DP), 2–60) and oligofructose/fructooligosaccharides (FOS) with shorter chains (DP, 2–8) [16]. In addition, neo-inulin and neo-levan types of fructans with internal glucose residues can also be found [17]. Benefits of fructans include gastrointestinal relief, increased calcium bioavailability, induction of apoptotic effects on colon cancer tumor cells, reduction of cholesterol and triglycerides, and modulation of the immune system [18,19,20,21].

#### 2.1.1. Inulin

**Figure 2:**
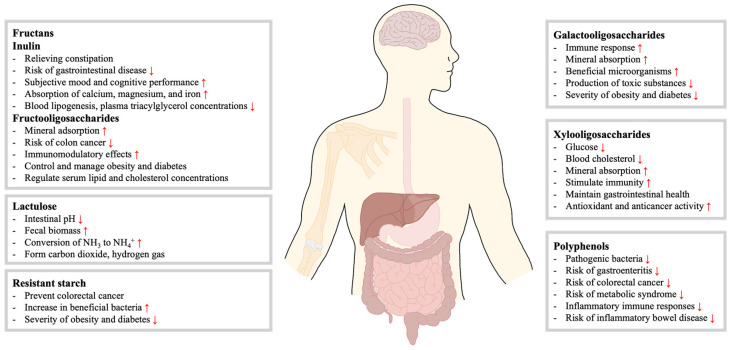
**Effects of prebiotics.** Fructans (inulin and fructooligosaccharides) improve gastrointestinal health, enhance mineral absorption, and lower blood lipids. Lactulose lowers intestinal pH and increases fecal biomass. Resistant starch prevents colorectal cancer and promotes beneficial bacteria. Galactooligosaccharides increase immune responses and beneficial microorganisms. Xylooligosaccharides lower glucose and cholesterol, improve mineral absorption, and stimulate immunity. Polyphenols reduce inflammation and the risk of inflammatory bowel disease.

Inulin is a water-soluble storage polysaccharide that belongs to a group of non-digestible carbohydrates called fructans. Inulin is generally recognized as safe (GRAS) in the United States and is abundant in around 36,000 plant species, with chicory root being the most concentrated source of inulin. In addition to chicory root, Jerusalem artichokes, dahlia tubers, yacon, asparagus, and leeks are natural sources of inulin [22]. Inulin contains (2→1) β-d-fructofuransonyl units (Fn) (n ≥ 60), typically with a (1↔2) α-d-glucopyranose (GFn) end group. The hydrolyzed form of inulin is known as oligofructose (n = 2–10) [23]. Inulin is a plant carbohydrate stored with a fructose motif connected by a β-(2-1)-d-fructosyl linkage. It is not digestible in the human small intestine because of its β-configuration of anomeric C2, but it can be fermented in the large intestine [24]. Almost 90% of inulin travels to the colon, where the bacteria digest it there [25]. Inulin’s health benefits include reducing blood lipogenesis and plasma triacylglycerol concentrations, relieving constipation, reducing the risk of gastrointestinal disease, and enhancing the absorption of calcium, magnesium, and iron (Figure 2) [22,26].

#### 2.1.2. Fructooligosaccharides (FOS)

**Figure 3:**
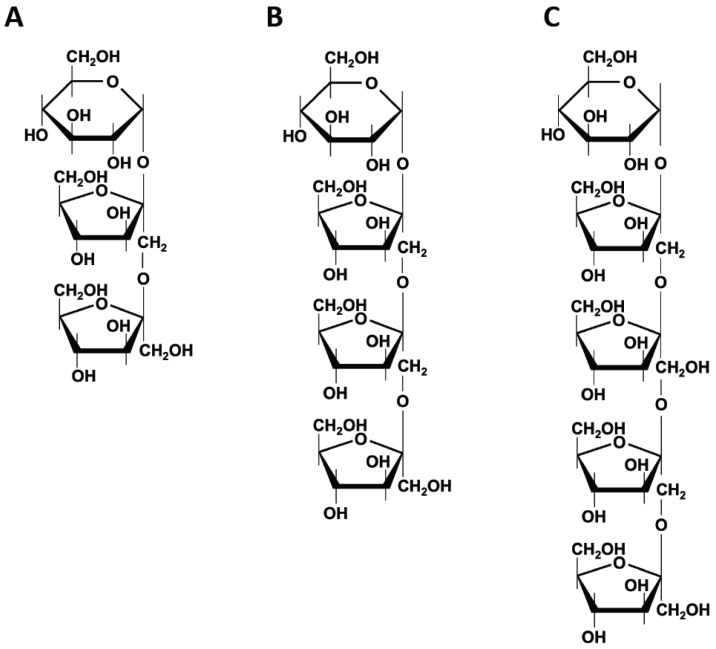
**Chemical structures of fructooligosaccharides (FOS).** FOS is a series of homologous oligosaccharides derived from sucrose, usually denoted by the chemical formula GFn: (**A**) 1-kestose (GF2); (**B**) nystose (GF3); (**C**) 1^F^-β-fructofuranosyl nystose (GF4).

FOS is the common name for fructose oligomers, generally understood as inulin-type oligosaccharides. They are a series of homologous oligosaccharides derived from sucrose, usually denoted by the chemical formula GFn, consisting of 1-kestose (GF2), nystose (GF3), and 1^F^-β-fructofuranosyl nystose (GF4) with two, three, and four fructosyl units bonded to the β-2,1 position of glucose, respectively (Figure 3) [27]. In general, FOS occurs naturally in some vegetables, such as onions, wheat, rye, shallots, tomatoes, and bananas, or it can be produced from sucrose or inulin by microbial enzymes, namely β-d-fructofuranosidase or fructosyltransferase, such as bacterial and fungal sources [28]. FOS consumption has been shown to prevent colon cancer, have immunomodulatory effects, control and manage obesity and diabetes, improve mineral adsorption, and regulate serum lipid and cholesterol concentrations (Figure 2) [29,30,31,32].

### 2.2. Galactooligosaccharides (GOS)

**Figure 4:**
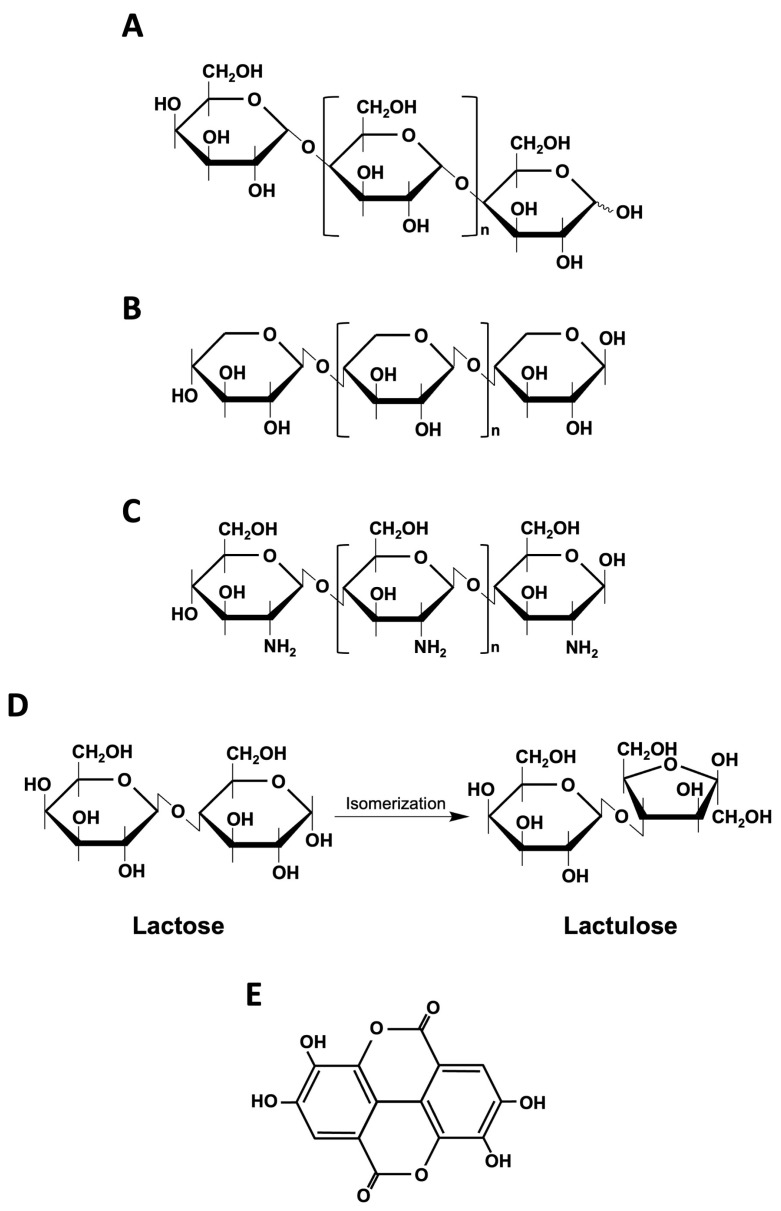
**Chemical structures of other types of prebiotics.** (**A**) Galactooligosaccharides (GOS) are non-digestible carbohydrates composed of 3–10 or more galactose molecules and a terminal glucose molecule. (**B**) Xylooligosaccharides (XOS) are linear oligosaccharides composed of d-xylose units linked by β-1, 4 glycosidic bonds. (**C**) Chitooligosaccharides (COS) are chitosan oligomers formed by the hydrolysis of chitosan. (**D**) Lactulose is produced through the isomerization of lactose. (**E**) Polyphenols are characterized by an aromatic ring containing one or more hydroxyl groups in their chemical structure.

GOS are non-digestible carbohydrates composed of 3–10 or more galactose molecules and a terminal glucose molecule (Figure 4A). GOS are produced through the catalysis of glycoside hydrolases, typically with lactose as a substrate, leading to a blend of GOS with varying polymerization levels. Several microbial glycoside hydrolases have been utilized for their manufacturing. Various biotechnological technologies, such as immobilizing or recombinant enzymes, have enhanced production [33]. GOS is also often used in milk-based products and infant formulas to mimic the effects of breast milk oligosaccharides because it is physiologically similar to breast milk [34]. The benefits of GOS include selective stimulation of beneficial microorganisms, reduced production of toxic substances, improved immune response, increased mineral absorption, and reduced severity of obesity and diabetes (Figure 2) [35].

### 2.3. Xylooligosaccharides (XOS)

XOS is a linear oligosaccharide with d-xylose units linked by β-1, 4 glycosidic bonds (Figure 4B). The DP of XOS ranges from 2 to 12 and is composed of xylobiose, xylotriose, xylotetraose, xylopentose, xylohexose, and xylohepatose [36]. The properties of XOS depend on its structure, the type of sugars present, and DP [37]. XOS is produced from xylan-containing lignocellulosic materials by chemical methods (e.g., autohydrolysis using water or steam or media catalyzed with externally added inorganic acids), a combination of chemical and enzymatic treatments, or direct enzymatic hydrolysis of sensitive substrates [38]. Consumption of XOS has been shown to have bifidogenic activity, reduce blood cholesterol, maintain gastrointestinal health, increase mineral absorption, stimulate immunity, have antioxidant and anticancer activity, and have the ability to reduce glucose (Figure 2) [39].

### 2.4. Chitooligosaccharides (COS)

COS refers to chitosan oligomers formed by the hydrolysis of chitosan, which contains somewhat unstable glycosidic bonds with a degree of polymerization (DP) of less than 20 and an average molecular weight (MW) of less than 3.9 kDa (Figure 4C) [40]. Compared to chitin and chitosan, which have high molecular weights, low solubility, and high viscosity, COS offer more advantages due to their solubility and lower molecular weight, enabling greater biological applications across various fields [41]. COS modulates the composition of the gut microbiota by increasing *Bacteroidetes*, decreasing *Proteobacteria* and *Actinobacteria*, and decreasing the *Firmicutes*/*Bacteroidetes* ratio [42].

### 2.5. Lactulose

**Figure 5:**
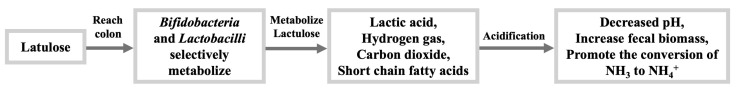
**Lactulose Metabolism and Effects as Prebiotics.** Lactulose is selectively metabolized by *Bifidobacteria* and *Lactobacilli* in the colon and has health effects through metabolites.

The traditional types of prebiotics mentioned above are all plant-derived prebiotics. Nevertheless, there are also artificially produced prebiotics. Lactulose is an artificial polysaccharide composed of galactose and fructose, generated via the isomerization of lactose (Figure 4D) [43]. Methods for producing lactulose include chemical, enzymatic, and electro-activation techniques [44]. Lactulose is resistant to hydrolysis by human small intestinal disaccharidases, so it reaches the colon intact, where *Bifidobacteria* and *Lactobacilli* selectively metabolize it to form lactic acid, carbon dioxide, hydrogen gas, and SCFAs, which increase fecal biomass and decrease pH (Figure 5) [45]. These acids biochemically draw water into the intestine and soften stool, so lactulose can be used as a laxative [46]. This acidification also promotes the conversion of NH_3_ to NH_4_^+^, which is not absorbed and excreted (Figure 2) [45].

### 2.6. Resistant Starch (RS)

Resistant starch (RS) refers to all starch and starch breakdown products that are not absorbed in the small intestine of healthy individuals [47]. After being digested in the small intestine, starch travels to the colon, where it is fermented by the gut microbiota. While many gut microbiota express α-amylases that can break down soluble starch, only a small number of gut microbiota can break down resistant starch, which is insoluble and highly resistant to digestion. The easily digestible starch fermented readily, producing acetate and lactate, while the resistant starch fermented much more slowly, producing acetate and butyrate [48]. RS is categorized into five categories. Resistant starch 1 is starch that is physically enclosed within the cell structure, hindering enzymatic breakdown. Resistant starch 2 is starch with a crystalline structure that is resistant to enzymatic action and loses this resistance when it undergoes gelatinization. Resistant starch 3 refers to retrograde starch, which occurs when amylose crystallizes and becomes resistant to enzymes, such as during the cooling of thermally gelatinized starch. Resistant starch 4 is a chemically modified starch that cannot be digested, while resistant starch 5 is a combination of amylose and lipids formed during starch processing. [49]. RS consumption has been shown to regulate gastrointestinal bacteria, prevent colorectal cancer, improve diabetes, and reduce obesity (Figure 2) [50,51].

### 2.7. Polyphenols

Polyphenols are secondary metabolites of plants. They range from simple phenolic molecules to complex polymers and are characterized by an aromatic ring containing one or more hydroxyl groups in their chemical structure (Figure 4D) [52]. Polyphenols are categorized into two main groups: flavonoids and non-flavonoids [53]. There are over 8000 polyphenols known to exist in plants, vegetables, and fruits, most of which reach the colon intact and are utilized by resident microorganisms [54]. The prebiotic effects of polyphenols are thought to be associated with the promotion of probiotics (e.g., *Bifidobacteriaceae* and *Lactobacillaceae*) or the reduction of pathogenic bacteria (e.g., *Escherichia coli, Clostridium perfringens*, and *Helicobacter pylori*), which reduces inflammatory immune responses and decreases the risk of gastroenteritis, colorectal cancer, metabolic syndrome, and inflammatory bowel disease (IBD) (Figure 2) [52,55]. In addition, polyphenols may promote β-oxidation, inhibit adipocyte differentiation, and counteract oxidative stress [56].

## 3. Prebiotics Modulate Gut Microbiota

Several studies have highlighted the importance of the relationship between the prebiotics and gut microbiota. Increasing the quantity of *Bifidobacterium* and *Lactobacillus* species, which ameliorate inflammatory bowel illness, aid digestion, lessen constipation, resist infection, and avoid traveler’s diarrhea, is one of the advantages of prebiotics [57]. Other studies have shown that prebiotics can influence SCFAs production, modulate the immune system, improve gut barrier function, reduce pathogenic bacteria populations, improve brain function and mineral bioavailability, reduce blood lipid levels, or affect insulin resistance, which is related to the cardiovascular system and more [58]. The fermentation of prebiotics by intestinal microbes produces several metabolites, of which SCFAs are a significant group [59,60]. In the microbiome, SCFAs are essential for balancing redox-equivalent production in the anaerobic environment of the gut [61]. SCFAs are organic fatty acids composed of 1–6 carbons. The main SCFAs are acetate (C2), propionate (C3), and butyrate (C4) [62]. SCFAs can act as a source of energy absorbed through the colon mucosa [63]. In addition to being an energy source, SCFAs have been shown to have many essential physiological functions, including maintaining luminal pH, inhibiting pathogen growth, influencing intestinal motility, and stimulating cancer cell apoptosis, thereby reducing colorectal cancer [64].

In several experiments, changes in the gut microbiota through the consumption of prebiotics have been observed. Treatment with inulin-type fructans (ITF prebiotics) increased *Bifidobacterium* and *Faecalibacterium prausnitzii*, which were negatively correlated with serum lipopolysaccharide levels. ITF prebiotics also decreased *Bacteroides intestinalis*, *Bacteroides vulgatus*, and *Propionibacterium*, which were associated with modest reductions in fat mass and plasma lactate and phosphatidylcholine levels. In this trial, ITF prebiotics induced subtle changes in the gut microbiota that could have important implications for several key metabolites associated with obesity or diabetes [65]. One experiment confirmed that the abundance of *Akkermansia_muciniphila* and *Ruminococcaceae_UCG_010*, which can effectively produce SCFAs, significantly increased due to the administration of GOS. Additionally, the quantity of *Bacteroides_vulgatus*, a symbiotic bacterium preventing *Vibrio cholerae* infection, also increased substantially. Such an augmentation of beneficial microbes indicates that prebiotics can promote gut microbiota health [66]. A prebiotic called inulin is known to influence gut microbiome regulation. Experiments have shown that this effect is related to the DP of inulin. In one mouse model study, high-fat diet mice were given inulin with DP ≤ 9 and DP ≥ 23 for 8 weeks. Inulin with a higher DP was found to have a more beneficial effect on liver damage by causing a greater increase in *Bacteroidetes* and a decrease in *Firmicutes* [67]. Prebiotics can also change the gut environment. The fermentation products of prebiotics are mostly acids, which reduce the pH in the gut [68]. It has been shown that a reduction in gut pH from 6.5 to 5.5 can contribute to changes in the composition and population of the gut microbiota [69]. At a pH of 5.5, there was an increase in butyrate production, and butyrate-producing bacteria such as *Roseburia* were more abundant. [70].

## 4. Influence of Prebiotics on Specific Health Conditions

### 4.1. Obesity

The prevalence of obesity has increased globally over the years, and its economic and health impacts are enormous. There is growing evidence that obesity is closely linked to changes in the composition and function of the gut microbiota. This evidence has led to the emergence of prebiotics as a possible solution to treat or prevent obesity. Characteristics of the gut microbiome of obesity include a high ratio of *Firmicutes/Bacteroidetes*, an abundance of gram negative bacteria, and low abundance and stability [71]. In an experiment using the TNO intestinal model (TIM-2) to identify gut microbiota by treating lactulose at different doses, it was seen that the proportion of *Bacteroides* increased compared to *Firmicutes* when 2 g of lactulose was treated [72]. A trial looked at the effect of prebiotics on childhood obesity by giving obese children and healthy children either oligofructose-enriched inulin (OI) or a placebo for 16 weeks. After 16 weeks, children who consumed OI showed a significant reduction in body weight z-score (3.1% reduction), body fat percentage (2.4% reduction), and trunk fat percentage (3.8% reduction) compared to children who consumed placebo. 16S rRNA sequencing also showed a significant increase in *Bifidobacterium* spp. and a decrease in *Bacteroides vulgatus* within the group that consumed OI [73]. In another experiment, compared to control mice fed a standard chow diet, a high-fat diet (HF) significantly decreased gut gram-negative and gram-positive bacteria, including *Bifidobacterium* spp. However, mice fed a high-fat oligofructose (HF-OFS) diet had a complete recovery in the amount of *Bifidobacteria*. Endotoxemia was significantly increased in HF-fed mice and normalized to control levels in HF-OFS-fed mice. These findings suggest that the gut microbiota contributes to the pathophysiological regulation of endotoxemia and creates the conditions for the development of diabetes and obesity. As a result, the consumption of prebiotics that support the growth of *Bifidobacteria* may help stabilize the gut microbiota, which is associated with the development of obesity [74].

### 4.2. Inflammatory Bowel Disease (IBD)

IBD is a collective term for a group of idiopathic bowel diseases, represented by ulcerative colitis and Crohn’s disease. IBD is a group of disorders associated with uncontrolled inflammation in the gastrointestinal tract. It is a chronic, recurrent disorder that has been shown to predispose to colon cancer later in life [75]. Crohn’s disease and ulcerative colitis are characterized by a severe bacterial imbalance in the gut microbiome, with an expansion of harmful taxa and depletion of beneficial members [76]. IBD patients have been shown to have increased numbers of *Proteobacteria* and *Actinobacteria* while decreasing amounts of dominant commensal bacteria such as *Firmicutes* and *Bacteroidetes* [77]. These decreases are associated with decreased levels of SCFAs in the feces of IBD patients [78]. Several trials have shown prebiotics to be effective in improving IBD. Inulin, one of the prebiotics, reduces the severity of dextran sodium sulfate (DSS) colitis when given orally [18]. In experiments comparing DSS-induced and non-DSS-induced mice, prebiotics potentially exerted beneficial anti-inflammatory effects through modulation of the gut microbiota, strengthening the intestinal barrier, and inhibiting IL-6/STAT3 signaling [79]. Using an intestinal epithelial model and a mouse model of stress-relapsing IBD to investigate the protective effects of inulin, it was observed that inulin significantly decreased the expression of pro-inflammatory cytokines (CSCL8/IL8 and TNFA) and increased MUC2 expression in intestinal epithelial cells. A significant protective effect of inulin consumption on IBD symptoms was demonstrated in colon samples through the downregulation of pro-inflammatory cytokines (IL6) and a decrease in serum inflammatory markers (IL-6, CALP). In addition, inulin intake decreased the expression of endoplasmic reticulum stress markers (CHOP, BiP) and increased SCFAs in cecal contents. The findings emphasize that inulin may improve stress-relapsing IBD symptoms by reducing inflammation, modulating microbiota composition, and alleviating endoplasmic reticulum stress. These results suggest the potential of inulin as a dietary treatment for stress-relapsing IBD [80].

### 4.3. Immune System

One of the beneficial effects of prebiotics is to stimulate the immune system, either directly or indirectly, by increasing the population of beneficial microorganisms or probiotics in the gut, especially lactic acid bacteria and *Bifidobacterial* [81]. In one experiment, inulin upregulated Th2-related immune genes (IL13, IL5) and suppressed Th1-related pro-inflammatory genes (IFNG, IL1A, and IL18) in the colon. Specifically, inulin increased the response by increasing the transcription of crucial Th2 and mucosal barrier genes and suppressing pro-inflammatory genes such as IFNG and CXCL9. They also confirmed through 16S rRNA sequencing of proximal colonic digestive samples that inulin supplementation decreased the abundance of bacterial phyla associated with inflammation, such as *Proteobacteria* and *Firmicutes*, while increasing *Actinobacteria* and *Bacteroidetes* [82]. In another study, two groups of male mice were fed a diet supplemented with 10% XOS or a control diet for 10 weeks. The expression of interleukin 1β (IL1β) and interferon γ (IFNγ) was significantly lower in the blood of XOS-fed mice compared to control mice. In vitro, they also found that treatment of blood with propionate, one of the SCFAs, significantly decreased the expression of IL1β, IFNγ, and interleukin 18 (IL18), supporting the hypothesis that increased production of SCFAs in the gut leads to systemic transport from the gut and downregulation of low-grade inflammatory cytokines [83]. Not only that, but prebiotics directly affect the immune system. Non-digestible oligosaccharides directly modulate host mucosal signaling, resulting in decreased responsiveness of intestinal epithelial cells to pathogen-induced mitogen-activated protein kinase and nuclear factor kappa B (NF-κB), modulating the host kinome and altering the host immune response without altering the gut microbiota [84].

### 4.4. Mental Health

It is also known that the diverse microbes found in the gastrointestinal tract can activate central nervous system signaling processes and neural pathways and contribute to the development of mental illnesses such as depression and anxiety [85]. This two-way communication between the brain and gut is called the gut-brain axis [86]. When 47 subjects were given a single dose of 5 g of inulin, researchers found that supplementation significantly improved subjective mood and cognitive performance. They also reported the most significant impact on episodic memory tasks, including improved accuracy and better recall performance on recognition memory tasks [87]. It has also been shown that a high prebiotic-rich whole plant food intake can reduce mood disorders, anxiety, and stress and improve sleep in nonclinical adults. Therefore, a high prebiotic diet may be a helpful strategy for increasing mental health in nonclinical populations [88]. It’s also a known fact that many psychiatric disorders stem from inflammation. Depression promotes inflammatory responses, and inflammation promotes depression and other neuropsychiatric disorders [89]. The ability of prebiotics to modulate the immune system mentioned above may alleviate this inflammation, which in turn may alleviate neuropsychiatric disorders [90].

### 4.5. Other Diseases

In addition to the ailments mentioned above, prebiotics positively affect many other diseases. FOS and GOS are essential for better density and resistance to bone wear and tear, as well as better absorption of calcium [91]. In studies in animal models, treatment with FOS resulted in higher serum alkaline phosphatase (ALP, a marker enzyme of bone formation used to diagnose skeletal and liver disease) levels and more resistant femurs. Increased bone density can increase bone strength, reducing fracture risk [92]. Chitooligosaccharides (COS) are used as an adjunctive therapy for diabetes, which has been shown to reduce blood sugar and blood lipids and is closely related to the gut microbiota. Studies in diabetic mice have shown that COS positively affects the composition of the gut microbiota. They found that COS lowered blood sugar by decreasing the amount of *Bacteroides* and increasing the amount of *Firmicutes* and *Actinobacteria* [93].

## 5. Synbiotics

In 2001, the Food and Agriculture Organization of the United Nations (FAO) and the WHO debated the emerging field of probiotics and defined the definition of probiotics: “Live microorganisms which when administered in adequate amount confer a health benefit on the host” [94,95]. The probiotic strains with proven health benefits are as follows: *Lactobacillus*, *Bifidobacterium*, *Saccharomyces*, *Enterococcus*, *Streptococcus*, *Pediococcus*, *Leuconostoc*, and *Bacillus* [96]. The main probiotic mechanisms of action include strengthening of the epithelial barrier, increased adhesion to the intestinal mucosa and subsequent inhibition of pathogen adhesion, competitive exclusion of pathogenic microorganisms, production of antimicrobial substances, and modulation of the immune system [97].

In 1995, Glenn R. Gibson and Marcel Roberfroid introduced the term synbiotic, which can be defined as a mixture of probiotics and prebiotics. It defined the survival and colonization of live microbial dietary supplements in the gastrointestinal tract as having a beneficial effect on the host by selectively stimulating the growth or activating the metabolism of one or a limited number of good and health-promoting bacteria and thus contributing to the well-being of the host [12]. Synbiotic is a nutritional supplement that synergistically combines probiotics and prebiotics, so synbiotics can enhance their beneficial effects. When two nutritional ingredients or supplements are administered together, the resulting positive effects generally follow one of three patterns: potentiation, synergy, and additive effects [98]. The primary purpose of such a combination is to improve the survival of probiotic microorganisms in the gastrointestinal tract. Synbiotics have both probiotic and prebiotic properties and were created to overcome the challenges that probiotics may face in the gastrointestinal tract. Probiotics beneficially affect the intestinal balance and form a protective barrier in the digestive tract. Prebiotics provide energy and nutrients to probiotic microorganisms, so the right combination of both ingredients in a single product can have a superior effect compared to either probiotics or prebiotics alone [99].

## 6. Market Potential of Prebiotics

According to a recent analysis, the global functional prebiotics ingredient market is estimated to be worth $4.5 billion in 2020 and is expected to reach more than $94 billion by 2026, considering a five-year CAGR of 8.7%. Among the established prebiotics, the market potential for FOS, inulin, and XOS in 2019–2020 was $2.37 billion, $1.4 billion, and $99 million, respectively, with annual growth rates of 10, 6.4, and 4.4%, respectively. The XOS market is expected to reach nearly $130 million by 2025, with a CAGR of 4.4% [100].

## 7. Perspective

The prebiotics mentioned in this review, such as fructans, galactooligosaccharides, xylooligosaccharides, chitooligosaccharides, lactulose, resistant starch, polyphenols, etc., are substances that have been shown to improve obesity, inflammatory bowel disease, mental health, and more. As people become more concerned about their health, more and more people are taking and seeking dietary supplements, such as prebiotics. For these reasons, as mentioned above, the market for prebiotics is growing, and many new prebiotics are being discovered. It is hoped that these discoveries will lead to more health benefits than those that have been found to date. However, more rigorous studies, such as well-designed major organ clinical trials, are needed to determine human health effects. However, it is expected that prebiotics can have a good effect on improving human health if taken properly and with sufficient knowledge.

## Figures and Tables

**Table 1:** Types of fructans.

Fructans	Linkage	Kestose Type
Inulin Fructooligosaccharides	β(2→1)	1-kestose
Neo-inulin	β(2→1)	6G-kestose
Levan	β(2→6)	6-kestose
Mixed levan (graminan)	β(2→1) β(2→6)	1-kestose 6-kestose
Neo-levan	β(2→1) β(2→6)	6G-kestose
